# Perspectives of English, Chinese, and Spanish-Speaking Safety-Net Patients on Clinician Computer Use: Qualitative Analysis

**DOI:** 10.2196/13131

**Published:** 2019-05-22

**Authors:** Elaine C Khoong, Roy Cherian, George Y Matta, Courtney R Lyles, Dean Schillinger, Neda Ratanawongsa

**Affiliations:** 1 Division of General Internal Medicine Department of Medicine at Zuckerberg San Francisco General Hospital University of California San Francisco San Francisco, CA United States; 2 UCSF Center for Vulnerable Populations at Zuckerberg San Francisco General Hospital University of California San Francisco San Francisco, CA United States; 3 School of Medicine Boston University Boston, MA United States

**Keywords:** vulnerable populations, electronic health records, attitude to computers, physician-patient relations, communication barriers

## Abstract

**Background:**

Safety-net systems serve patients with limited health literacy and limited English proficiency (LEP) who face communication barriers. However, little is known about how diverse safety-net patients feel about increasing clinician electronic health record (EHR) use.

**Objective:**

The aim of this study was to better understand how safety-net patients, including those with LEP, view clinician EHR use.

**Methods:**

We conducted focus groups in English, Spanish, and Cantonese (N=37) to elicit patient perspectives on how clinicians use EHRs during clinic visits. Using a grounded theory approach, we coded transcripts to identify key themes.

**Results:**

Across multiple language groups, participants accepted multitasking and silent clinician EHR use if focused on their care. However, participants desired more screen share and eye contact, especially when demonstrating physical concerns. All participants, including LEP participants, wanted clinicians to include them in EHR use.

**Conclusions:**

Linguistically diverse patients accept the value of EHR use during outpatient visits but desire more eye contact, verbal warnings before EHR use, and screen-sharing. Safety-net health systems should support clinicians in completing EHR-related tasks during the visit using patient-centered strategies for all patients.

## Introduction

Electronic health record (EHR) system use during outpatient visits affects patient-provider communication, clinician workload, and clinician well-being [1-4]. Enabled by federal incentives, American safety-net clinics, publicly funded facilities providing care for socioeconomically disadvantaged populations, have experienced tremendous growth in EHR implementation. EHRs affect the care experience of safety-net patients, exacerbating communication barriers related to limited health literacy or limited English proficiency (LEP) [5]. However, delaying EHR use until after an office visit may increase stress in a workforce at risk for burnout or medical errors (from inaccurate charting or forgetting to place orders) [6-9].

Clinicians engage with EHRs by multitasking while talking with patients or using them silently, potentially diverting their attention from patients [10]. Little is known about how linguistically diverse safety-net patients feel about clinician EHR use during clinic visits. Prior work has suggested that LEP populations may have different perspectives, experiencing higher amounts of EHR use but perceiving greater benefits to that use [11]. This short study aims to further explore how safety-net patients, including those with LEP, feel about clinician EHR use.

## Methods

### Setting and Recruitment

We conducted focus groups with ethnically and linguistically diverse patients to elicit perspectives on clinician EHR use during outpatient visits. Through posted flyers and in-person recruitment at 7 primary and specialty care clinic waiting rooms (family medicine, adult primary care, obstetrics or gynecology, diabetes, anticoagulation, cardiology, and gastroenterology or hepatology), we identified eligible patients: (1) English, Cantonese, or Spanish-speaking, (2) adults, and (3) receiving primary or specialty care from an urban safety-net hospital. This safety-net system cares for a population that is 16% African-American, 23% Asian, and 37% Latinx and insured predominantly by Medicaid (58%) or Medicare (22%) [12]. A prior study found nearly 50% of patients have inadequate health literacy [13]. There are 24/7 interpretation services via phone interpreters with video or in-person interpreters available during business hours. This system utilizes an Office of National Coordinator (ONC) certified EHR [14]. We collected sociodemographic characteristics by telephone, including use of validated questions to assess English proficiency and health literacy [15,16]. We provided participants US $35 for participation.

### Data Sources and Collection

The focus group guide (Multimedia Appendix 1) was created by the study team using an iterative process based on analysis of prior studies. We used consensus to ensure questions would be accurately translated before inclusion in the final focus group guide, which was translated into Chinese and Spanish by fluent, bilingual team members. It included questions on perceptions about overall communication, clinician communication, EHR use during visits, multitasking and silent EHR use, and preferences for EHR use. We used videos of reenacted examples [10] to demonstrate silent versus multitasking EHR use to help participants distinguish between these types of use. In Cantonese, there is no single word to convey the idea of *multi-tasking* so we felt demonstration of these concepts through videos was necessary.

We conducted 6 (3 English, 2 Cantonese, 1 Spanish) in-person 90-min focus groups in patients’ preferred languages from November 2017 to January 2018. A total of 2 Spanish focus groups were planned, but 1 focus group became a one-on-one interview when all other participants did not attend the focus group. The Spanish and English focus groups were conducted by a bilingual research team member; the Cantonese focus groups were conducted by a different bilingual team member. We acquired verbal and written consent from participants before participation. The focus groups were audio-recorded then transcribed into English for analysis. The research team contained Spanish and Cantonese speakers who could consult the audio files if participants’ meaning was unclear in English transcripts.

### Data Analysis

Using a grounded theory approach [17,18], we (EK, GM, NR) independently coded the same 3 transcripts (1 from each language), then through consensus developed a codebook (Multimedia Appendix 2) that encompassed themes represented in these transcripts. One team member (GM) then applied this codebook to all transcripts using ATLAS.ti 7.0 (ATLAS.ti Scientific Software Development GmbH); no additional themes were identified during this process, suggesting primary thematic saturation [19]. We triangulated results with primary care clinicians through interactive presentations and determined findings most likely to impact clinical practice.

This study was approved by the University of California San Francisco Institutional Review Board.

## Results

### Participant Characteristics

There were 37 participants (Table 1). Of the 37, 11 were Cantonese-speaking and 5 Spanish-speaking. Mean age was 54, and 57% (21/37) were women. Nearly half (17/37, 46%) reported limited health literacy, 41% (15/37) reported infrequent personal computer use, and 41% (15/37) reported poor or fair health. Cantonese-speaking participants were more likely to report limited health literacy and feel their primary care providers (PCPs) do not know them well.

**Table 1:** Demographic characteristics of participants in focus groups (N=37).

Characteristic		All	English (n=21)	Cantonese (n=11)	Spanish (n=5)
Age (years), mean (SD)		54 (10.4)	55 (10.3)	57 (6.2)	46 (14.9)
Women, n (%)		21 (57)	11 (52)	7 (64)	3 (60)
**Race, n (%)**					
	White	10 (27)	10 (48)	0	0
	Black or African-American	6 (16)	6 (29)	0	0
	Latinx	6 (16)	1 (5)	0	5 (100)
	Asian or Pacific Islander	11 (30)	0	11 (100)	0
	American Indian or Alaskan Native	1 (3)	1 (5)	0	0
	More than one	2 (5)	2 (10)	0	0
	Other	1 (3)	1 (5)	0	0
Limited English proficiency^a^, n (%)		15 (41)	0	11 (100)	4 (80)^b^
Limited health literacy^c^, n (%)		17 (46)	2 (10)	11 (100)	4 (80)^b^
Native language, limited health literacy^d^, n (%)		—^e^	—	7 (64)	1 (20)^b^
Poor or fair health, n (%)		15 (41)	4 (19)	8 (73)	3 (60)^b^
Primary care provider knows me well, n (%)		17 (46)	12 (57)	2 (18)	3 (60)^b^
Uses computer never or less than monthly, n (%)		15 (41)	6 (29)	7 (64)	2 (40)^b^

^a^Participants who reported speaking English less than “well.”

^b^One participant declined to answer this question.

^c^Somewhat, a little bit, or not at all confident “filling out medical forms by yourself.”

^d^Somewhat, a little bit, or not at all confident “filling out medical forms by yourself” if in native language (Spanish or Chinese).

^e^Reported only for participants with limited English proficiency.

### Perspectives on Electronic Health Record Use

Table 2 contains themes and representative quotes. Themes were consistent across language groups and classified into 2 categories: perspectives on EHR use and strategies for patient-centered EHR use.

Participants generally accepted EHR use, recognizing that its use assisted with care. This acceptance was conditional on the assumption that EHR use was focused on their care. Participants felt similarly about multitasking and silent EHR use, reporting that each type of use was appropriate during different parts of the visit.

### Suggestions for Patent-Centered Electronic Health Record Use

Despite the general acceptance of silent, multitasking, and frequent EHR use, participants had suggestions for how clinicians could exhibit more patient-centered EHR use.

Participants uniformly desired more eye contact during EHR use. In particular, some felt computer use is inappropriate when patients are attempting to show physical concerns:

If I have a sore throat, don’t just put it in the computer—look!

English-speaking participant

All participants reported a desire for clinicians to communicate what they were doing in the computer. In particular, they asked clinicians to provide a warning (ie, signaling) before transitioning to silent EHR use.

As part of this desire for transparency about EHR use, participants—including LEP participants—desired screen sharing:

When the doctor is typing...can I look at it at the same time?

Cantonese-speaking participant 1

Like show a big TV screen.

Cantonese-speaking participant 2

Yes. When the doctor is typing, then I can see it.

Cantonese-speaking participant 1

**Table 2:** Key themes and example quotes.

Themes		Example quotes
**Perspectives on EHR^a^** **use**		
	Patient-focused electronic health record use is acceptable	Spanish-speaking participant: “I think technology is important not just for keeping patient records but also for finding information. If a patient wants to know about a medication he’s been prescribed, it’s all right there” *;* English-speaking participant: “It hasn’t been an issue when she’s—she’s doing it [using the computer]. And, I’m still the only person in the room. So, I’m still getting 100% of our attention.”
	Silent and multitasking EHR use is expected and generally accepted	Cantonese-speaking participant: “When the doctor is ordering medications, he/she can stop talking to the patient, and concentrate on ordering the medication. When doctor is supposed to do one thing, then he/she should do that one thing” *;* English-speaking participant: “I think that doctors have always had to multi-task. Throughout history, there’s always a thousand instruments they’re having to deal with, they had to deal with many different cases...they’re always multi-tasking. Computer is just a tool.”
**Strategies for patient-centered EHR use**		
	More eye contact is desired	Cantonese-speaking participant: “If he (doctor) only looks at the medical record, not face the patient and only looks at the computer, then there is a distance between the doctor and patient” *;* English-speaking participant: “Look at me when you’re talking to me instead of looking at the screen and typing.”
	Limit computer use while patients show a physical concern to clinicians	Cantonese-speaking participant: “For some illness, you have to look at it to see it...For example, nails problem. You have to look at it to see it. If you only look at the computer...then you won’t know it” *;* English-speaking participant: “if a doctor was on the computer asking me ‘hey how’s your pain from 1 to 10’...I would want for her to… look closely at me...at my leg how I can move it, stuff like that...I wouldn’t want my doctor being on the computer while that doctor was examining, giving me a physical...”
	Communicate the purpose of computer use	English-speaking participant: “Just communicate. Communicate, communicate, communicate...’I got to take a minute and type this. I want to make sure it’s right.’ And then read it back or whatever; Cantonese-speaking participant: “Before the doctor orders medications, he/she should let you know: ‘This is what is your situation, I am going to prescribe this medication for you.’...So are you worried? You’re not worried.” Spanish-speaking participant: “When he’s on the computer he should explain what he’s talking about.”
	Share the screen	Spanish-speaking participant: “...seeing the lab results. It’s fantastic...I can look, too, and ask ‘What about my anemia? What does that red line mean?’ So, then she explains to me and tells me what we need to do...”; English-speaking participant: “I think the most important thing is just knowing that the patient would like to be a part of what’s going on, on the computer.”

^a^EHR: electronic health record.

## Discussion

### Principal Findings

In this short report, we found consensus among linguistically diverse safety-net patients on several themes regarding clinician EHR use. Previously, when this setting employed an EHR that did not meet ONC-certification requirements [14], we found that non-English-speaking patients reported more computer use but less concern that PCPs listened less carefully because of computer use; moreover, Asian patients had higher odds of reporting that computers helped PCPs remember patient concerns [11]. The findings of this study, conducted 3 to 4 years after implementation of a more comprehensive, certified EHR, suggest that safety-net patients across multiple languages experience frequent clinician EHR use but recognize its value to their care even if clinicians multitask or use EHRs silently during visits.

Despite acceptance of EHR use, participants provided suggestions for clinicians to improve the patient experience during EHR use. Safety-net patients—including limited health literate and LEP patients whom clinicians may not expect to read the EHR—wanted clinicians to be transparent about EHR use and even engage them in the process of EHR use. In triangulating our findings with clinicians, we found clinicians felt reassured that patients accepted exam room EHR use and surprised that LEP patients wanted to be included in what clinicians were doing on the computer.

### Recommendations for Patient-Centered Electronic Health Record Use

These findings in safety-net patients augment existing recommendations for patient-centered EHR use [20-23]:

1. You can use EHRs during visits. Consider asking patients how they feel about EHR use, as participants in this study accepted EHR use if focused on their care, consistent with prior literature [4,24]. This may ease clinician concerns about in-room charting and reduce the burden of after-hours charting [3].
2. Tell and show patients, including LEP patients, what you are doing, and offer a warning before transitioning to silent EHR use. As patients frequently initiate conversations during silence, you should signal to patients if you need to focus temporar­­­ily to complete an EHR task safely [10,25-27].
3. Connect with patients by maximizing eye contact and limiting silent EHR use [22,28-30]. Cease computer use when discussing emotional concerns, as previously recommended [31,32], but also when patients are showing physical concerns on their body.

### Study Limitations and Strengths

This study is limited by a small sample size within each language from a single setting, lack of information about patient-clinician language concordance, inability to report a response rate, and use of a single individual to code transcripts after developing themes through consensus. Strengths of this study are a diverse, safety-net population and recruitment from both outpatient primary care and specialty clinics.

### Practice Implications

As educators develop communications curricula for patient-centered EHR use, these findings and other perspectives from diverse patients should inform the content [23,33,34]. Future efforts should investigate multilevel interventions to increase adoption of patient-centered EHR use strategies, including computer (EHR user interface or content), patient (activation or empowerment), environment (redesign or reposition equipment in rooms), and policy level (incentives) interventions.

### Conclusions

Linguistically diverse safety-net patients accept the prevalence and utility of EHR use during outpatient visits, if focused on their care. However, there continues to be room for improvement for clinicians to adopt patient-centered strategies, including eye contact, signaling EHR use, and screen-sharing with safety-net patients.

## Appendix

Multimedia Appendix 1 Focus group interview guide.

Multimedia Appendix 2 Codebook.

## Abbreviations

AHRQ: Agency for Healthcare Research and Quality
EHR: electronic health record
LEP: limited English proficiency
NIH: National Institutes of Health
ONC: Office of National Coordinator
PCP: primary care provider
