# Supplementary material for: Perspectives of English, Chinese, and Spanish-Speaking Safety-Net Patients on Clinician Computer Use: Qualitative Analysis
Source: J Med Internet Res. 2019 May 22;21(5):e13131. doi: 10.2196/13131 (PMC6549473; doi:10.2196/13131)
Supplement: Multimedia Appendix 1 [file jmir_v21i5e13131_app1.pdf]

FOCUS GROUP QUESTION GUIDE

***Introduction*:**

Hi every one, thank you for joining our study about patient-provider communication. I'm your facilitator, ______________________, and I am a research analyst with Dr. Neda Ratanawongsa. I'll be helped today by __________.
We'd like to get your point of view and hear your experiences about the way your doctors use the computer. When we use the word doctor, we are also including nurse practitioners or physician assistants who provide your medical care.

You were selected to participate because you received care within the last year at one of the clinics at the Zuckerberg San Francisco General Hospital.

Setting the guidelines:

We will be recording this session to review what has been said. Since we are recording, we ask that one person speak at a time. Please also speak up.

We want you to feel safe sharing your feelings. We will keep your information confidential. We'll remove your names from the transcripts and any research reports. We also ask that you keep what you hear today confidential. Please don't tell someone else's story outside of this room.

We also want to hear from everyone. Please share how you feel even if it's different from what others say. There are no right or wrong answers—different experiences are important to us and everyone's experience is unique. We'll try to make sure that everyone gets a chance to speak. Please help us by showing respect for each other's opinions.

Our roles, as facilitators, will be to ask about 8 questions within the next hour and a half. We'll try to keep the conversation flowing and encourage people to speak up.

Does anyone have any questions?

Questions:

To begin, let's go around the group. Please say your name and if you have other rules or guidelines you'd like to add let us know.

Thank you.

How easy is it for you to talk with your doctors during your visits? To be clear, we are focusing on the time you and your provider are in the exam room together.

PROBES:

- **What makes it easier?**
- **What makes it harder?**
- **Has any of that changed over the last few years?**

Now, in general, **how much do doctors use computers when you see them?**

PROBES:

- **What do you think about that?**
- **What do others think? Do others feel differently?**

Now, we'd like to hear from each of you about how your doctors use the computer when they see you.

PROBES:

- **How does the computer help you during your visit? We'd like to hear examples.**
- **How does the computer make things harder for you during your visit? We'd like to hear examples.**

  Optional: **What did you do when that happened?**

Now I'm going to show you some ways that doctors use computers. We've seen that most doctors use the computers while talking or listening to patients at the same time.

- **What do you think about the doctor trying to talk with you while using the computer?**

Here are a couple of examples of doctors talking while using the computer. [Show video clips.]

2MultitaskingEHR10_11.mp4

Two videos:
Video 1: Clinician reviews medication list and asks patient questions while maintaining gaze at screen
Video 2: Clinician looks at patient to asks questions then asks patient if they can use the computer and invites patient to look at screen with them

PROBES:

- **What do you think?**
- **What do you like or not like?**
- **What do you experience?**

PROBE:

- **Some people call this multitasking, what do you think?**

At other times, they use the computer silently. [Show video clips.]

SilentEHR10_11.mp4

Two silent EHR use videos:
Video 1: Clinician asks patient for time to review the EHR
Video 2: Clinician is asking patient a question and in mid-sentence fades into silence

What do you think of how the doctors are using the computers in these examples and how do they relate to your experiences?

PROBES:

- **What do you think about the doctor focusing on you and then on the computer at different times?**
- **What would help make this better for you?**
- **Think of a time when you felt the doctors weren't listening to you because of the computer. What did you do? What do you wish you did?**

Some doctors say that it's hard to write prescriptions or order tests on the computer while talking or listening. They are worried about making mistakes. **If your doctor felt that way, what should they say to you?**

PROBES:

- **What do you do while the doctor is working on the computer in silence?**
- **Here are some ways doctors may ask for time to use the computer.** [Show video of providers verbalizing need for silent EHR time, with researcher-generated strategies to using that time.]

Strategies.mp4

Two videos on strategies for patient actions during silence:
Video 1: Clinician asks patient to review patient education materials while clinician reviews the EHR
Video 2: Clinician asks patient to identify three strategies to improve glucose management while clinician reviews the EHR

- **What would you think if your doctor said that to you?**
- **If the doctor said what they were typing out loud or showed you what they were typing, how would that feel?**

Now the computer sometimes includes information about when you last got your medicines. We wanted to show you an example of this and see what you thought of this.

VIDEO4

What other advice do you have for doctors about how they should use computers around patients?

Of everything that we’ve talked about today, what do you think is the most important thing that has been said?

Closing:

Alright, we’re done! We appreciate everything everyone has shared. Thank you for taking the time to help us today. We really want to help both patients and their doctors, and this is going to be very helpful. Thank you again.
