# Supplementary material for: Perspectives of English, Chinese, and Spanish-Speaking Safety-Net Patients on Clinician Computer Use: Qualitative Analysis
Source: J Med Internet Res. 2019 May 22;21(5):e13131. doi: 10.2196/13131 (PMC6549473; doi:10.2196/13131)
Supplement: Multimedia Appendix 2 [file jmir_v21i5e13131_app2.docx]

**Appendix 2.** Codebook

| Perceptions of Electronic Health Record (EHR) |
| --- |
| Intensity of EHR Use |
| Feelings about EHR Use |
| Perceptions of EHR and its Function |
|  |
| Types of EHR Use |
| EHR Silent Use |
| EHR  Multitasking |
|  |
| Current Use Patterns |
| How Providers Interact w Patients & EHR |
| How Providers Use EHR |
|  |
| Suggestions |
| Suggestions for Provider EHR Use |
| Most Important Strategies for Provider EHR Use |
|  |
| **ADDITIONAL THEMES (NOT FOCUS OF THIS MANUSCRIPT’S ANALYSIS)** |
| Overall Perceptions of Providers (including Non-EHR Related Patient-Provider Relationship and Communication) |
| Overall Perceptions of Quality of Care (including Systems Issues) |
| Suggestions / Strategies for How Patients Can Interact with EHR |
| Perceptions of Patient Portal |
| Patient Non-EHR Related Needs and/or Priorities |
| Participant Self-Identified Traits |
|  |
